# Integrating sociocultural theories to inform public health response: unique relationships between moral foundations, cultural cognition worldviews, and COVID-19 attitudes and behaviors

**DOI:** 10.3389/fpubh.2025.1606229

**Published:** 2025-12-19

**Authors:** Christopher Wolsko, Elizabeth Marino

**Affiliations:** Laboratory for the American Conversation, Oregon State University - Cascades, Bend, OR, United States

**Keywords:** cultural worldview, COVID-19, Moral Foundations Theory, vaccine attitudes, individualism

## Abstract

Key variables from Moral Foundations Theory and the Cultural Theory of Risk were examined in relationship to COVID-19 attitudes and behaviors. Two surveys were conducted with unvaccinated U. S. adults (*n* = 1,025) that assessed five moral foundations (*care*, *fairness*, *authority*, *loyalty*, and *purity*), two dimensions of Cultural Cognition Worldviews (*individualism-communitarianism* and *hierarchy-egalitarianism*), COVID-19 vaccination likelihood and related attitudes (including measures of perceived risk, protective behavior, and trust), political orientation, and demographic characteristics. The individualism-communitarianism scale, derived from the Cultural Theory of Risk, was the most impactful predictor across surveys. When controlling for responses to all other measures, participants who placed greater emphasis on individualism tended to report a lower likelihood of getting a COVID-19 vaccination, to perceive a lower level of risk from COVID-19, and to express greater distrust in the safety of vaccines developed by the government. Many other moral and cultural worldview dimensions were uniquely predictive of COVID-19 attitudes as well, while political orientation and demographic characteristics were generally weak or non-significant in multiple regression models. Findings underscore the sociocultural foundations of health behavior.

## 1 Introduction

Across a wide array of health-related contexts, research demonstrates that individuals’ attitudes and behaviors are not primarily driven by rational, information-based processing, but are rather an outcome of complex interrelationships between heuristics (1, 2), emotions (3, 4), cognitive biases (5, 6), social identities (7, 8), and cultural worldviews (9, 10). Conclusions from investigations into vaccination messaging and behavior are consistent with this picture, demonstrating that fact-based appeals are fairly ineffective (7, 11). Communication strategies are more impactful when they emphasize shared social identities and/or values between the source and the intended audience (12–16).

Research on vaccination, as well as behavior in response to other salient risks, such as climate change, illustrates the ways in which people interpret information through their cultural and ideological frameworks, aligning their beliefs and decisions with ingroup identities and important social values (17, 18). For example, certain individuals may downplay the risks of climate change and resist public health measures like vaccination, not necessarily due to lack of knowledge, but because the authoritative argument that is chronically presented for the risk, or for the solution, implicitly or explicitly threatens their social identity or worldview (19, 20). The lower levels of vaccine uptake that have been observed among political conservatives, including for COVID-19 (21, 22), may be interpreted in this light – where it is not concern about health and safety that is being rejected, but rather the ideological and moral tone of the prevailing health care discourse, in which getting vaccinated signifies acting like a political outgroup member, and thus being unfaithful to one’s ingroup values.

The present investigation examines the unique predictive effects of key social value orientations on COVID-19 vaccine intentions and related attitudes. While much of the contemporary “culture war” discourse in the U. S. frames polarization on health behavior and policy preferences in terms of political orientation, here, we assess the more nuanced contributions of two leading theoretical frameworks on human values and perceptions of risk: Moral Foundations Theory and the Cultural Theory of Risk.

Briefly, Moral Foundations Theory (23, 24) argues that individuals ground their moral judgments in a set of five core foundations: care, fairness, loyalty, authority, and purity, which vary in importance between cultural groups. Sporadic research has applied this framework to vaccination attitudes, and certain moral foundations have been found to be significantly associated with acceptance or hesitancy. For example, increased vaccine hesitancy has been associated with higher purity concerns (7, 16, 25, 26). Stronger endorsement of the authority dimension has been associated with both hesitancy (26) and with greater acceptance (25). Likewise, care has been associated with greater hesitancy (25) and greater acceptance (26), and displayed no relationship with vaccination behavior as well (16).

The Cultural Theory of Risk (27) argues that individuals perceive and respond to risks based on their cultural worldviews and social structures rather than on the basis of objective calculations. The theory categorizes cultural worldviews along two key dimensions: *grid*, which concerns the degree to which individuals’ roles and rules are highly structured or prescribed by society; and *group*, which concerns the degree to which individuals are highly identified with and committed to specific social groups. Kahan (19) operationalized these theoretical dimensions in the form of two cultural cognition worldview scales: individualism-communitarianism (assessing *group* by contrasting the preference for personal freedom and choice with greater regulation by societal, and especially governmental, structures) and hierarchy-egalitarianism (assessing *grid* by contrasting social stratification with a preference for greater equality). As with Moral Foundations Theory, these dimensions have received some limited attention in terms of their power to predict vaccination attitudes. Greater emphasis on individualism and hierarchy has been associated with lower perceived COVID-19 risk and severity (28–31), lower support for COVID-19 public health responses (32), as well as more perceived COVID-19 vaccination risks, less perceived COVID-19 vaccination benefits, and lower support for COVID-19 mandatory vaccination (33). COVID-19 mitigation measures have received greater support from those who favor egalitarianism, communitarianism, and hierarchy (34).

These frameworks intersect in the sense that certain moral foundations align with specific cultural worldviews. For instance, there is considerable similarity between the fairness moral dimension and the egalitarian worldview, and between the morality of authority and a hierarchical worldview (35). Also, an individualist worldview tends to prioritize liberty and resist the structures imposed in the “binding” moralities of loyalty and authority; while egalitarians may emphasize care and fairness.

To assess the unique contributions of these moral foundations and cultural worldviews to our understanding of individuals’ vaccination behavior, it is essential to examine the predictive effects of both simultaneously, and we can find no study in the literature to date that has done so. Even separately, their predictive power has been examined in few studies, which is surprising given the relatively strong associations that have been found. Below, we describe the results of two surveys in which we examined the relationships between moral foundations, cultural worldviews, and the likelihood of COVID-19 vaccination and other related attitudes.

Consistent with prior work, we hypothesized that there would be greater vaccine hesitancy among those who supported more of an individualistic worldview, and less of a communitarian worldview. The individualism-communitarianism dimension investigated here is similar to the classic individualism–collectivism dimension (36), which has been shown to predict a wide range of health behaviors (37), including those related to COVID-19 (51, 38). In the present context, we expected that individualists’ greater preoccupation with liberty and freedom of choice would predict lower vaccine uptake, and the emphasis on the collective good would predict higher vaccine uptake.

Consistent with prior work on moral foundations, we hypothesized that a greater emphasis on the moral of purity would predict greater vaccine hesitancy, due to concerns about the safety and artificiality of vaccines [e.g., (7, 16)]. Care has tended not to reliably predict vaccination behavior, but has been associated with greater support for COVID-19 mitigation behaviors (39), and should theoretically be correlated with health behavior that demonstrates care and protection for the self and others. Beyond these predictions, our analyses are exploratory, given the inconsistent relationships with vaccine attitudes reviewed above.

## 2 Materials and methods

### 2.1 Survey 1

Two separate surveys were conducted during the winter of 2020–2021, just as the public rollout of the COVID-19 vaccines in the U. S. we’re beginning to take shape. We conducted these two surveys a few months apart in order to assess the stability of relationships between key variables during this time when public option was quite dynamic. Additionally, the end of the second survey also served as a focus group recruitment tool for a separate qualitative investigation into COVID-19 vaccine attitudes not discussed in the present manuscript. All participants were sampled from the Amazon MTurk system and completed one of two short 15-min surveys in exchange for $1.00. Below, the materials and methods for each survey are described separately.

#### 2.1.1 Participants

Survey 1 was conducted on November 3, 2020, and had 511 participants. This survey was conducted prior to the public availability of a COVID-19 vaccine in the U. S. The sample had a majority of male respondents (60.6% male, 39.3% female); was majority Caucasian (75.1% White, 14.5% Black/African American, 4.7% Latino/Hispanic, 4.9% Asian American, 0.2% Pacific Islander; 0.6% American Indian / Alaska Native); diverse in age (*M* = 38.07, *SD* = 11.59; range: 19 to 70 years old); diverse in highest educational attainment (1.4%, some high school; 5.9%, high school diploma or GED; 14.9%, some college or associates degree; 61.1%, bachelor’s degree; 16.8%, master’s degree or higher); and fairly representative of the U. S. population in household income (7.1%, less than $25,000; 29.2% from $25,000 to $49,999; 33.5% from $50,000 to $74,999; 20.4% from $75,000 to $99,999; 9.9%, $100,000 or more).

#### 2.1.2 Survey measures

Participants responded to the Moral Foundations Questionnaire and the Culture Cognition Worldview Scales, as well as assessments of vaccine likelihood, perceived COVID-19 risk, frequency of engagement in COVID-19 protective behaviors, trust in information sources, political orientation, and demographic characteristics. Key variable names are italicized in the descriptions that follow.

#### 2.1.3 Moral foundations questionnaire

Participants responded to the 30-item version of the *Moral Foundations Questionnaire* (MFQ-30) (24). Individuals were instructed to indicate their level of agreement with each of 15 moral statements (e.g., “Compassion for those who are suffering is the most crucial virtue.”) on a 7-point scale, ranging from 1, *strongly disagree*, to 7, *strongly agree*; and to indicate how relevant each of 15 considerations is when deciding whether something is right or wrong (e.g., “Whether or not someone showed a lack of respect for authority.”) on a 6-point scale ranging from 0, *not at all relevant*, to 5, *extremely relevant*. Within each set of 15 items, 3 items assess each of the five moral dimensions, resulting in 6 items per dimension. A mean was calculated for each set of 6 items, resulting in a score for each participant that reflected their level of *care*, *fairness*, *loyalty*, *authority*, and *purity*, with higher scores indicating more emphasis place on the given moral dimension.^1^

#### 2.1.4 Cultural cognition worldview scales

Next, participants completed the long-form of the Cultural Cognition Worldview Scales (CCWS) (40). Individuals were instructed to indicate their level of agreement with a set of 17 items assessing the group dimension of cultural cognition (e.g., “Too many people today expect society to do things for them that they should be doing for themselves.”) and 13 items assessing the grid dimension (“Parents should encourage young boys to be more sensitive and less rough and tough.”) on 7-point scales, ranging from 1, *strongly disagree*, to 7, *strongly agree*. Each participant received a *Individualism-Communitarianism* score, which was calculated as the average level of agreement across all group items (reverse-scoring, where appropriate), with higher numbers reflecting a greater preference for individualism over communitarianism and a *Hierarchy-Egalitarianism* score, which was calculated as the average level of agreement across all grid items (reverse-scoring, where appropriate), with higher number reflecting a preference for hierarchy over egalitarianism.

#### 2.1.5 Vaccine likelihood

Participants were asked, “Assuming you will have free access to it, how likely are you to get a coronavirus (COVID-19) vaccine as soon as the Food and Drug Administration (FDA) approves one for the general population?” Responses were provided on a 4-point scale, ranging from 1, *will not get the vaccine*, to 4, *very likely that I’ll get the vaccine*. Participants were assigned a *vaccine likelihood* score based on their response to the question, with higher numbers reflecting greater likelihood.

#### 2.1.6 Assessment of COVID-19 risk

Participants indicated their level of agreement with 5 statements that addressed level of concern with COVID risks, including, “The coronavirus poses a major threat to the public” and “Very few people in the country are likely to actually get sick from the virus.” Responses were provided on a 7-point scale, ranging from 1, *strongly disagree*, to 7, *strongly agree*. A mean across all five items was calculated for each participant (reverse scoring, where appropriate), with higher numbers reflecting greater perception of *COVID-19 risk*.

#### 2.1.7 Protective behavior

Participants indicated the frequency with which they had engaged on each of 14 different protective behaviors over the prior month, including “Worn a mask in indoor public spaces” and “Avoided indoor gatherings with people outside my household.” Responses were provided on a 5-point scale, ranging from 1, *never*, to 5, *always, or almost always*. Participants were assigned a *protective behavior* score, calculated as the mean across all items, with higher numbers reflecting a higher frequency of protective behavior.

#### 2.1.8 Trust in information sources

Participants were asked about the extent to which they trusted information about the coronavirus from each of the following sources: scientists, physicians, the Centers for Disease Control, the Food and Drug Administration, and your local county health department. Reponses were provided on a 5-point scale, ranging from 1, *do not trust at all*, to 5, *trust a great deal*. Item responses were consistently and positively intercorrelated, so we combined them into a single index of *trust in information*, taking the mean of all responses to the five items, with higher numbers indicating greater trust. The reliability of the index was acceptably high (*α* = 0.79).

#### 2.1.9 Demographics

Finally, participants responded to a series of demographic questions, including gender, age, education, annual income, race/ethnicity, and political orientation. Descriptions of these variables were presented above, with the exception of political orientation, which was assessed by asking participants to indicate the degree to which they identified as more liberal or more conservative. Responses were obtained on a 7-point scale (1 = *extremely liberal*; 2 = *moderately liberal*, 3 = *slightly liberal*; 4 = *moderate*; 5 = *slightly conservative*; 6 = *moderately conservative*; 7 = *extremely conservative*), and each individual received a *political orientation* score based on their response to this item.

### 2.2 Survey 2

#### 2.2.1 Participants

Survey 2 was conducted on February 23, 2021, and had 514 participants. This survey was conducted approximately 2 months after the first COVID-19 vaccine was available in the U. S., and participants were instructed to complete the survey only if they had not been vaccinated and not made plans or a formal appointment to do so. The demographics were similar to Survey 1, except for a slightly lower relative percentage of male respondents (54.5% male, 44.0% female). The majority were Caucasian (72.5% White, 9.2% Black/African American, 6.8% Latino/Hispanic, 10.7% Asian American, 0.2% Pacific Islander; 0.6% American Indian / Alaska Native); diverse in age (*M* = 38.43, SD = 11.76; range: 18 to 83 years old); diverse in highest educational attainment (0.8%, some high school; 11.1%, high school diploma or GED; 20.8%, some college or associates degree; 49.8%, bachelor’s degree; 18.4%, master’s degree or higher); and representative of the U. S. population in household income (11.0%, less than $25,000; 25.8% from $25,000 to $49,999; 24.5% from $50,000 to $74,999; 20.7% from $75,000 to $99,999; 18.0%, $100,000 or more).

#### 2.2.2 Survey measures

Participants in Survey 2 responded to the same measures of Moral Foundations, Culture Cognition Worldviews, vaccine likelihood, political orientation and demographic characteristics, as described above for Survey 1. Responses to the *vaccine likelihood* variable were provided on a 6-point scale, ranging from 1, *will definitely not get the vaccine*, to 6, *I am certain that I’ll get the vaccine* (rather than the 4-point scale utilized in Survey 1).

Additionally, participants in Survey 2 indicated their level of agreement with the following statement: “I do not trust the government to make sure the COVID vaccines are safe and effective.” Responses were provided on a 7-point scale, ranging from 1, *strongly disagree*, to 7, *strongly agree*. Each participant was assigned a *distrust in vaccine safety* score reflecting their response on this item, with higher numbers indicating greater distrust.

## 3 Results

### 3.1 Correlations between moral foundations, cultural cognitions, and COVID variables

We first examined the zero-order correlations between our sociocultural variables (the five moral foundations scales and the two cultural cognition scales) and our COVID-related variables. Correlation coefficients from Survey 1 are presented in Table 1 and those from Survey 2 are presented in Table 2. In Survey 1, participants who indicated they were more likely to get vaccinated tended to score higher on *care* and *fairness*, and lower on *individualism-communitarianism* and *hierarchy-egalitarianism* (reflecting a greater preference for communitarianism and egalitarianism). In Survey 2, participants who indicated they were more likely to get vaccinated tended to score higher on *fairness*, lower on *loyalty*, *authority*, and *purity*, and lower on *individualism-communitarianism* and *hierarchy-egalitarianism* (reflecting a greater preference for communitarianism and egalitarianism).

**Table 1:** Zero-order correlations between moral foundations, cultural cognitions, and COVID-19 attitudes (survey 1).

	Care	Justice	Loyalty	Authority	Purity	Ind-Comm	Hier-Egal	Vaccine likelihood	COVID-19 risk	Protective behavior	Trust in information
Fairness	0.77***										
Loyalty	0.35***	0.32***									
Authority	0.37***	0.40***	0.83***								
Purity	0.29***	0.29***	0.79***	0.82***							
Ind-Comm	−0.04	−0.06	0.43***	0.47***	0.40***						
Hier-Egal	−0.25***	−0.27***	0.41***	0.41***	0.43***	0.73***					
Vaccine Likelihood	0.25***	0.22***	0.03	0.02	−0.01	−0.24***	−0.26***				
COVID-19 Risk	0.10*	0.06	−0.55***	−0.51***	−0.52***	−0.63***	−0.63***	0.23***			
Protective Behavior	0.55***	0.59***	0.13**	0.18***	0.11*	−0.21***	−0.39***	0.39***	0.35***		
Trust in Information	0.43***	0.45***	0.24***	0.21***	0.15***	−0.15***	−0.31***	0.32***	0.12**	0.58***	

Correlation is significant at *p < 0.05, **p < 0.01, ***p < 0.001.

**Table 2:** Zero-order correlations between moral foundations, cultural cognitions, and COVID-19 attitudes (survey 2).

	Care	Justice	Loyalty	Authority	Purity	Ind-Comm	Hier-Egal	Vaccine likelihood	Distrust in vaccine safety
Fairness	0.73***								
Loyalty	0.27***	0.22***							
Authority	0.24***	0.19***	0.82***						
Purity	0.28***	0.16***	0.73***	0.80***					
Ind-Comm	−0.25***	−0.26***	0.35***	0.42***	0.35***				
Hier-Egal	−0.36***	−0.39***	0.39***	0.46***	0.41***	0.77***			
Vaccine Likelihood	0.06	0.13**	−0.13**	−0.17***	−0.24***	−0.40***	−0.35***		
Distrust in Vaccine Safety	0.06	−0.02	0.39***	0.39***	0.46***	0.39***	0.39***	−0.59***	

Correlation is significant at *p < 0.05, **p < 0.01, ***p < 0.001.

The other COVID-related variables were also significantly related to many of the moral foundations and cultural cognition subscales. In Survey 1, lower perceived *COVID-19 risk* was most strongly associated with higher *loyalty*, *authority*, and *purity*, and by a greater emphasis on individualism and hierarchy. Also in Survey 1, engaging in more protective behavior and trusting mainstream COVID-19 information sources were most strongly related to more concern with *care* and *fairness*. In Survey 2, *distrust in vaccine safety* was most associated with higher *loyalty*, *authority*, and *purity*, and by a greater emphasis on individualism and hierarchy.

### 3.2 Correlations between demographics and COVID variables

We also examined relationships between participants’ demographic characteristics and COVID attitudes. Relationships with age, income, education, and political orientation are presented in Table 3 (Survey 1) and Table 4 (Survey 2). Age, income, and education were fairly weakly and inconsistently associated with COVID attitudes. On the other hand, *political orientation* was associated with lower *vaccine likelihood* (Surveys 1 and 2), lower perceived *COVID-19 risk* (Survey 1), lower frequency of *protective behavior* (Survey 1), and greater *distrust in vaccine safety* (Survey 2).

**Table 3:** Zero-order correlations between demographics and COVID-19 attitudes (survey 1).

	Vaccine likelihood	COVID-19 risk	Protective behavior	Trust in information
Age	0.15***	−0.04	0.09*	0.09*
Income	−0.02	−0.10*	−0.12**	−0.08
Education	0.05	−0.17***	0.00	0.07
Political Orientation	−0.12**	−0.39***	−0.15***	−0.07

Correlation is significant at *p < 0.05, **p < 0.01, ***p < 0.001.

**Table 4:** Zero-order correlations between demographics and COVID-19 attitudes (survey 2).

	Vaccine likelihood	Distrust in vaccine safety
Age	0.02	−0.12**
Income	0.07	0.01
Education	0.17***	−0.03
Political Orientation	−0.32***	0.37***

Correlation is significant at *p < 0.05, **p < 0.01, ***p < 0.001.

Relationships between COVID attitudes and gender and race/ethnicity were sporadic. The means for all COVID attitudes as a function of these demographics are presented in Table 5 (Survey 1) and Table 6 (Survey 2). In Survey 1, female respondents indicated they felt more at risk from COVID than did male respondents, *t* (508) = 2.43, *p* = 0.008. In Survey 2, male respondents indicated they distrusted the government to make vaccines safe and effective more than female respondents, *t* (503) = 2.13, *p* = 0.017. No other comparisons were significant.

**Table 5:** Means for COVID-19 attitudes by gender and race/ethnicity (survey 1).

	*n*	Vaccine likelihood	COVID-19 risk	Protective behavior	Trust in information
Male	308	2.71	3.68	3.95	3.83
Female	200	2.65	3.98	3.96	3.79
Caucasian/White American	382	2.66	3.84	3.90	3.77
Black/African American	74	2.70	3.42	4.09	3.95
Latino/Hispanic	24	2.96	3.69	4.19	4.24
Asian American	25	2.52	4.38	4.07	3.71

**Table 6:** Means for COVID-19 attitudes by gender and race/ethnicity (survey 2).

	*n*	Vaccine likelihood	Distrust in vaccine safety
Male	280	4.10	3.68
Female	226	3.99	3.30
Caucasian/White American	382	4.08	3.34
Black/African American	74	3.51	4.26
Latino/Hispanic	24	4.11	3.02
Asian American	25	4.45	4.17

Relationships between race/ethnicity and COVID attitudes were examined within each survey via one-way ANOVA with post-hoc Tukey’s HSD test for multiple between-group comparisons. In Survey 1, Asian American respondents reported being more likely to get a COVID vaccine than did African American respondents, *p* = 0.038; and Latino respondents reported trusting COVID information from government and science more than did White American participants, *p* = 0.010. In Survey 2, Asian American respondents reported being more likely to get a COVID vaccine that did African American respondents, *p* = 0.011. Also in Survey 2, Whie respondents reported feeling more trust in vaccine safety than did Asian American participants (*p* = 0.022) and African American participants (*p* = 0.016). Additionally, Latino respondents reported more trust in vaccine safety than did White American participants (*p* = 0.030) and Asian American participants (*p* = 0.041).

### 3.3 Unique predictive effects of cultural cognitions and moral foundations on COVID attitudes

As all of our key sociocultural variables were intercorrelated with one another (sometimes very strongly), it was clearly necessary to examine the unique contributions of each to predicting our COVID-related measures. To do so, we conducted a series of stepwise linear regressions to examine the unique predictors of each of our COVID variables: Survey 1 – *vaccine likelihood*, *COVID-19 risk*, *protective behavior*, and *trust in information*, and Survey 2 – *vaccine likelihood* and *distrust in vaccine safety*. For each of these variables, the predictors in the regression models included scores on all five dimensions of Moral Foundations Theory (*care*, *fairness*, *loyalty*, *authority*, and *purity*), scores on both of the Cultural Cognition Worldview Scales (*individualism-communitarianism* and *hierarchy-egalitarianism*), and all demographics, which included age (continuous), gender (male vs. female), annual household income (continuous), highest educational attainment (continuous), ethnicity (white/Caucasian vs. ethnic minority), and political orientation (continuous). We report statistics on the significant terms in the final model in the stepwise procedure.

### 3.4 Survey 1 models

In the model predicting *vaccine likelihood*, *care*, *individualism-communitarianism*, and age emerged as significant predictors (*R^2^* = 0.13). Participants who indicated they would be more likely to get vaccinated tended to express a higher degree of the *care* moral dimension, *b* = 0.31, *t* (502) = 5.20, *p* < 0.001; and a greater concern with communitarianism over individualism, *b* = −0.30, *t* (502) = −5.78, *p* < 0.001; and were older, *b* = 0.01, *t* (502) = 3.19, *p* = 0.001.

In the model predicting *COVID-19 risk*, *care*, *individualism-communitarianism*, *loyalty*, *care*, *hierarchy-egalitarianism*, and *purity* all emerged as significant predictors (*R^2^* = 0.57). Individuals who perceived a higher level of risk from COVID-19 tended to place greater value on communitarianism over individualism, *b* = −0.54, *t* (504) = −7.11, *p* < 0.001; lower on loyalty, *b* = −0.38, *t* (504) = −5.98, *p* < 0.001; higher value on care, *b* = 0.39, *t* (504) = 5.56, *p* < 0.001; a greater emphasis on egalitarianism over hierarchy, *b* = −0.25, *t* (504) = −3.43, *p* < 0.001; and a lower concern with purity, *b* = −0.15, *t* (504) = −2.75, *p* = 0.006.

In the model predicting *protective behavior*, *care*, *justice*, *hierarchy-egalitarianism*, *ethnicity*, and *income* were significant predictors (*R^2^* = 0.44). Participants who engaged in greater levels of *protective behavior* tended to express a stronger concern for the *care* moral dimension, *b* = 0.19, *t* (504) = 3.62, *p* < 0.001; a stronger concern for the *fairness* moral dimension, *b* = 0.31, *t* (504) = 5.46, *p* < 0.001; a greater emphasis on egalitarianism over hierarchy, *b* = −0.22, *t* (504) = −6.70, *p* < 0.001; a lower income, *b* = −0.04, *t* (504) = −2.30, *p* < 0.001; and tended to be ethnic minority, relative to white people, *b* = −0.07, *t* (504) = −2.66, *p* = 0.008.

In the model predicting *trust in information*, *fairness*, *hierarchy-egalitarianism,* and *loyalty* were significant predictors (*R^2^* = 0.30). Participants who expressed greater trust had higher fairness concerns, *b* = 0.28, *t* (504) = 5.68, *p* < 0.001, a greater emphasis on egalitarianism over hierarchy, *b* = −0.30, *t* (504) = −7.95, *p* < 0.001, and higher loyalty concerns, *b* = 0.21, *t* (504) = 6.54, *p* < 0.001.

### 3.5 Survey 2 models

In the model predicting vaccine likelihood, *individualism-communitarianism*, *purity*, *authority*, *education*, *income*, and *political orientation* (*R^2^* = 0.23). Participants indicated a greater likelihood of getting the vaccine if they tended to place greater value on communitarianism over individualism, *b* = −0.50, *t* (499) = −6.55, *p* < 0.001; less value on purity, *b* = −0.57, *t* (499) = −3.80, *p* < 0.001; placed more value on authority, *b* = 0.57, *t* (499) = 3.30, *p* < 0.001; were more educated, *b* = 0.30, *t* (499) = 3.91, *p* < 0.001; had higher income, *b* = 0.10, *t* (499) = 2.15, *p* = 0.032; and were more politically liberal, *b* = −0.12, *t* (499) = −2.50, *p* < 0.001.

In the model predicting distrust in vaccine safety (*R^2^* = 0.30), individuals tended to distrust the safety of vaccines developed by the government if they were higher in *purity*, *b* = 0.88, *t* (499) = 8.21, *p* < 0.001; higher in individualism over communitarianism, *b* = 0.38, *t* (499) = 4.73, *p* < 0.001; younger, *b* = −0.03, *t* (499) = −4.52, *p* < 0.001; and more conservative, *b* = 0.10, *t* (499) = 2.03, *p* = 0.043.

## 4 Discussion

Our findings indicate that Moral Foundations Theory and the Cultural Theory of Risk have considerable importance as social value frameworks that help us understand individuals’ vaccination intentions and related attitudes. The individualism-communitarianism scale was arguably the most impactful predictor across surveys and COVID-19-related variables in the present investigation. Participants who placed greater emphasis on individualism tended to report a lower likelihood of getting a COVID-19 vaccination, perceive a lower level of risk from COVID-19, and express greater distrust in the safety of vaccines developed by the government. The emphasis on freedom from government interference in the *individualism-communitarianism* scale items (e.g., “*It’s not the government’s business to try to protect people from themselves”*) concurs with the tenor of popular anti-government and anti-vaccine discourse at the time of this survey. While the cultural cognition scales emphasize a more politicized freedom from government intervention more than do traditional scales assessing individualism, these findings dovetail with existing research demonstrating that preoccupations with individual liberty may inhibit vaccine uptake advised by the more collectivist orientation of public health institutions [e.g., (38)].

In a democratic state, respecting individuals’ liberty while also creating solidarity to serve the needs of a collective in times of crisis is a perennial public health challenge. Pre-existent sociopolitical polarization in the U. S. was exacerbated by the COVID-19 pandemic, and concern about the role of government in public health continues into the present. As Jay Bhattacharya, newly appointed head of the National Institutes of Health in the U. S., recently remarked in an interview with *The Wall Street Journal*, many people now see “the scientific establishment as essentially an authoritarian power sitting over them, rather than as a force for good” (41). This is a social reality that needs to be seriously acknowledged and addressed in order to better serve the public health needs of a diverse populace.

Other dimensions of morality and cultural cognition were uniquely predictive of vaccine attitudes as well. Those who placed greater emphasis on the morality of care tended to report greater likelihood of getting vaccinated, perceived COVID-19 as more dangerous, and engaged in a higher frequency of protective behavior. Higher moral concern with fairness was uniquely associated with engaging in more protective behavior and greater trust in official sources of COVID-19-related information. Purity was associated with less likely vaccination and more distrust of the governmental vaccine development process; and a preference for egalitarianism was associated with a tendency to perceive COVID-19 as more dangerous, trust information from official sources more, and engage in more protective behavior. Unique effects of political orientation and other demographics tended to be weak and inconsistent, indicating that the moral values and cultural worldviews were far more powerful predictors.

A significant limitation of this study was the utilization of samples from the Amazon MTurk system. MTurk is an online labor market that has been widely utilized by survey researchers in psychology and other social sciences, and while samples are generally demographically diverse and display strong psychometric properties (e.g., test–retest reliability, experimental replication) (42, 43), representativeness and data quality have been increasing concerns with the acceleration of online human-subjects research (52). Additionally, MTurk samples have been shown to be limited in their representativeness of the health behaviors of the general population [e.g., (44, 45)].

The current investigation adds to an emerging empirical record of strong relationships between moral foundations, cultural worldviews, and vaccine-related attitudes (7, 16, 25, 26, 28–31). This work underscores how critical it is to realize that communicating about vaccines and other health care issues actually means communicating about deeply held values and complex cultural constructions of risk. There are multiple subcultures to engage with in any public health issue, each with their own worldviews and moral imperatives. Attending to these sociocultural factors through outreach and tailored messaging campaigns is not just helpful, but arguably, foundational.

As pro-environmental discourses have been largely reliant on appeals to the moral dimensions of care and fairness (46, 47), so too has vaccination messaging been focused on invoking such altruistic and egalitarian perspectives (48–50). The great diversity of values that were uniquely predictive of COVID-19-related attitudes in the current investigation, taken together with the findings of others’ work reviewed here, suggests that it would be impactful to appeal to a much wider range of values in public health communications. Which values to focus on will depend on the cultural contexts in which salient public health concerns are currently embedded. In the highly polarized sociopolitical environment of the U. S. at the moment, achieving a balance and reconciliation between communitarian and individualist worldviews could not be more critical.

## Data Availability

The raw data supporting the conclusions of this article will be made available by the authors, without undue reservation.

## Footnotes

1 The agree-disagree items were assessed on a 7-point scale (with a neutral / neither agree nor disagree option) unlike the original version of the scale that has no neutral point. Thus, in taking the means of items from separate scales, we first standardized all responses and then took the mean of the 6 z-scores for each participant.
